# Mesmerism in India, and Its Practical Application in Surgery and Medicine

**Published:** 1846-10

**Authors:** 


### Art. XII.

*Mesmerism in India, and its practical Application in Surgery and Medicine.* By James Esdaile, m.d.—*London,* 1846. 12mo, pp. 287.

In our article on Mesmerism, eighteen months ago, we entered into an extended discussion concerning the validity of mesmeric facts, and the theory of *animal magnetism;* avoiding, however, all detailed examination of mesmeric doctrines in their application to the treatment of disease. We passed lightly over this latter topic, because, in dealing with the subject as a matter of evidence affecting its reality, we had consumed the whole of our space at that time available; yet we expressed a hope that a future opportunity would arise for supplying the omission in question. This opportunity, the appearance of Dr. Esdaile's book now presents to us.

Dr. Esdaile occupies the post of civil assistant surgeon to one of the hospitals in Bengal; a situation which furnishes him with ample means for testing the value of controverted modes of treatment. The experience which he has given to the world in this book is, assuredly, not a little startling: it has relation to the practical application of mesmerism in both surgery and medicine. The statements which he has put forth, we shall summarily notice; and then we shall submit a few remarks upon their credibility, and upon the consequence which, we think, should flow from them.

Our author informs us that, prior to his own experiments, he was almost altogether unacquainted with the subject; having neither read mesmeric books, nor witnessed experiments in the practice of others. The first case which came beneath his notice was quite accidental; and this circumstance is cited by himself as enhancing the credibility of the accounts which he publishes. He states, that had he selected for his first essay one whom he should have deemed to be an appropriate subject, he would most probably have taken "some highly sensitive female of a nervous temperament and excitable imagination, who desired to submit to the supposed influence." He observes that in the instance in question, however, he was guided by no previously adopted theory, but was determined purely by accident in the choice of his subject, who happened to be a Hindoo felon, of the hangman caste, condemned to labour in irons on the roads. Though somewhat lengthy, we will insert the details of this experiment in Dr. Esdaile's own words; previously directing the reader's attention to certain facts upon which the author himself lays great stress, as dissipating, in his own judgment, every source of deception and fallacy; these are: 1st, the purely accidental and unpremeditated nature of the experiment; 2d, all want of consent between the parties; 3d, the operator's want of belief in his own power, and slight knowledge of the subject; 4th, the absolute ignorance of the patient, who could not possibly have heard of mesmerism; and 5th, the impossibility deduced from the foregoing considerations, of the patient's imitating the mesmeric phenomena.

"*First experiment.* Mádhab Kaurá, a hog-dealer, condemned to seven years' imprisonment, with labour on the roads, in irons, for wounding a man so as to endanger his life, has got a double hydrocele. He was ordered to be taken from the jail to the charity hospital, to be operated upon.

"April 4th. The water was drawn off one side of the scrotum, and two drachms of the usual cor. sub. injection were thrown in. On feeling the pain from the injection he threw his head over the back of the chair, and pressed his hands along the course of the spermatic cords, closing his eyelids firmly, and making the grimaces of a man in pain. Seeing him suffering in this way, I turned to the native sub-assistant surgeon, an *élève* of the medical college, and asked him if he had ever seen mesmerism. He said that he had seen it tried at the medical college, but without effect. Upon which I remarked, 'I have a great mind to try it on this man; but as I never saw it practised, and know it only from reading, I shall probably not succeed.' The man continued in the position described, I placed his knees between mine, and began to pass my hands slowly over his face, at the distance of an inch, and carried them down to the pit of his stomach. This was continued for half an hour before he was spoken to, and when questioned at the end of this time his answers were quite sensible and coherent.

"He was ordered to remain quiet, and the passes were continued for a quarter of an hour longer—still no sensible effect. Being now tired (thermometer 85°), I gave it up in despair, and declared it to be a failure. While I rested myself the man remained quiet, and made fewer grimaces, and when ordered to open his eyes he said there was smoke in the room. This roused my attention, and tempted me to persevere. I now breathed on his head, and carried my hands from the back of his head over his face and down to the epigastrium, where I pressed them united. The first time this was done he took his hands off his groins, and pressed them both firmly down upon mine, drew a long breath, and said, 'I was his father and mother, and had given him life again.' The same process was persevered in, and in about an hour he began to gape, said he must sleep, that his senses were gone; and his replies became incoherent. He opened his eyes when ordered, but said he only saw smoke, and could distinguish no one; his eyes were quite lustreless, and the lids were opened heavily. All appearance of pain now disappeared; his hands were crossed on his breast, instead of being pressed on the groins, and his countenance showed the most perfect repose. He now took no notice of our questions; and I called loudly on him by name without attracting any notice.

"I now pinched him without disturbing him, and then asking for a pin in English, I desired my assistant to watch him narrowly, and drove it into the small of his back. It produced no effect whatever; and my assistant repeated it at intervals in different places as uselessly. His back had continued to arch more backwards latterly, and he was now in a state of 'opisthotonos;' the nape of his neck resting on the sharp back of the chair, and his breech on the edge of it. Being now satisfied that we had got something extraordinary, I went over to the Kutcherry, and begged Mr. Russell, the judge, and Mr. Money, the collector, to come and see what had been done, as I wanted the presence of intelligent witnesses in what remained to do. We found him in the position I had left him in, and no hallooing in his ears could attract his attention. Fire was then applied to his knee, without his shrinking in the least; and liquor ammoniæ, that brought tears into our eyes in a moment, was inhaled for some minutes without causing an eyelid to quiver. This seemed to have revived him a little, as he moved his head shortly afterwards, and I asked him if he wanted to drink; he only gaped in reply, and I took the opportunity to give, slowly, a mixture of ammonia so strong that I could not bear to taste it; this he drank like milk, and gaped for more. As the 'experimentum crucis,' I lifted his head, and placed his face, which was directed to the ceiling all this time, in front of a full light; opened his eyes, one after the other, *but without producing any effect upon the iris;* his eyes were exactly like an amaurotic person's, and all noticed their lack-lustre appearance. We were all now convinced that total insensibility of all the senses existed, and I ordered him to be placed on a mattress on the floor, and not to be disturbed till I returned. It was now one o'clock, the process having commenced at 11 a.m.

"I returned at three o'clock, and was vexed to find that he had awoke, and been carried back to the jail hospital. The native doctor of the jail had come in, and on hearing that the Sahibs could not awake the patient, he set about doing so, and succeeded by throwing water on his face."

Dr. Esdaile maintains that an especial value attaches to early experiments in any new subject of investigation. In reference to his own trials of mesmerism in the first instance, he likens his position to that of the chemist who, having heard that a new elementary substance had been discovered by a certain process, sets his own apparatus to work in the way prescribed, and is rewarded by obtaining the same results as the first discoverer; these, Dr. Esdaile observes, surprise the experimenter even, who feels confident that he only relates what he actually saw, and that he is not seduced by previous theory and prepossession of mind to interpret appearances in support of a foregone conclusion.

Having, in the manner just stated, become satisfied that there was a truth in mesmerism, Dr. Esdaile informs us that he lost no time in turning it to practical account; and the success which he recounts surpasses all previous records of this nature. If we may credit the publication now before us, it would appear that almost every attempt to reduce a patient to the mesmeric condition was more or less successful. Indeed, the statements which are made regarding the facility of inducing insensibility prior to surgical operations are, to say the least, very remarkable, we may say, not a little suspicious. Within the period of eight months, viz. from May 1845 to January of the present year, Dr. Esdaile reports himself to have performed no less than seventy-three painless operations; and some of these were of the most formidable character, as may be inferred from the following synopsis furnished in a prefatory Report of Mesmeric Facts:

"*A return showing the number of painless surgical operations performed at Hooghly, during the last eight months.* Arm amputated, 1. Breast ditto, 1. Tumour extracted from the upper jaw, 1. Scirrhus testium extirpated, 2. Penis amputated, 2. Contracted knees straightened, 3. Ditto arms, 3. Operations for cataract, 3. Large tumour in the groin cut off, 1. Operations for hydrocele, 7. Ditto dropsy, 2. Actual cautery applied to a sore, 1. Muriatic acid ditto, 2. Unhealthy sores pared down, 7. Abscesses opened, 5. Sinus, six inches long, laid open, 1. Heel flayed, 1. End of thumb cut off, 1. Teeth extracted, 3. Gum cut away, 1. Prepuce cut off, 3. Piles ditto, 1. Great toe nails cut out by the roots, 5. Seton introduced from ankle to knee, 1. Large tumour on leg removed, 1. Scrotal tumours, weighing from 8 lb. to 80 lb., removed 17, painless, 14.—Operations, 73."

This statement, we conceive, involves an amount of success in the production of the mesmeric coma, far beyond anything that is recorded in this country, or indeed in Europe. Dr. Esdaile assigns a purely hypothetical, we may say visionary cause for this. He attributes its comparative rarity in Europe to the "influence not being at once sufficiently concentrated on the patient, by transmitting it to his brain from all the organs of the operator, and through every channel by which it can be communicated." He says that, in India, with due patience and sustained attention, coma, in a tolerably extensive field of experience, can be produced daily, so that insensibility to the surgeon's knife will ensue; the process adopted by himself is set forth in the subjoined extract:

"Desire the patient to lie down and compose himself to sleep, taking care, if you wish to operate, that he does not know your intention: this object may be gained by saying it is only a trial; for fear and expectation are destructive to the physical impression required. Bring the crown of the patient's head to the end of the bed, and seat yourself so as to be able to bring your face into contact with his, and extend your hands to the pit of the stomach, when it is wished; make the room dark, enjoin quiet, and then shutting your patient's eyes, begin to pass both your hands, in the shape of claws, slowly, within an inch of the surface, from the back of the head to the pit of the stomach; dwelling for several minutes over the eyes, nose, and mouth, and then passing down each side of the neck, go downwards to the pit of the stomach, keeping your hands suspended there for some time. Repeat this process steadily for a quarter of an hour, breathing gently on the head and eyes all the time. The longitudinal passes may then be advantageously terminated, by placing both hands gently, but firmly, on the pit of the stomach and sides; the perspiration and saliva seem also to aid the effect on the system.

"It is better not to test the patient's condition by speaking to him, but by gently trying if the cataleptic tendency exists in the arms. If the arms remain fixed in any position they are left in, and require some force to remove them out of every new position, the process has been successful; the patient may soon after be called upon by name, and pricked, and if he does not awake the operation may be proceeded with."

It would carry us beyond the limited space which we can give to the present notice, were we to furnish any detailed account of the various operations performed by Dr. Esdaile; it is not, however, necessary to do so. It has been seen in what way he was led to experiment in the first instance, and the particulars of the subsequent cases, in which the knife was employed without eliciting signs of sensibility, do not differ from the generality of this class of facts, exemplified in our former article upon this subject. We may, in the sequel however, have occasion to refer to some particulars of individual cases, in briefly discussing the credibility of these narratives.

We will now refer to the statements which are made concerning the alleged therapeutical action of mesmerism. Dr. Esdaile says, that it is useful in chronic inflammation, by gently stimulating the nerves and capillary vessels to more healthy action; and, with respect to functional derangements of the nervous system, he hopes that, at length, in this mesmeric agency, we have obtained a *direct* nervous remedy. The medical cases adapted for the use of mesmerism did not very often occur in our author's practice; because, says he, "the labouring poor do not usually resort to medical advice for nervous diseases till they are past cure;" he yet furnishes accounts of a number of them which he considers to have been beneficially influenced by the new remedy. The particulars correspond very much with the accounts which are given by other writers on mesmeric therapeutics. They are, on the whole, very unsatisfactory, like their predecessors. Not a few of them leave ample room for doubt, first, as to the reality of the disease treated; and, secondly, admitting their reality, as to the cure being effected by the alleged means.

The following is a summary of the medical cases said to have been treated successfully by mesmerism, within the before-mentioned period of eight months:

"*A return of medical cases cured by mesmerism during the last eight months.*

Nervous headache, 3 cured by one trance. Tic-doloureux, 1 ditto. Nervousness, and lameness from rheumatism of 2½ years' standing, 1 by chronic treatment. Spasmodic colic, 1 by one trance. Acute inflammation of the eye, 1 by repeated trances in 24 hours. Chronic ditto, 1 by chronic treatment. Acute inflammation of testes, 1 by repeated trances in 36 hours. Convulsions, 1 by one trance. Lameness from rheumatism, 2 by chronic treatment. Lumbago, 1 by general and local mesmerising for a week. Sciatica, 1 ditto. Pain in crural nerve, 1 ditto. Palsy of one arm, 1 ditto for a month. Ditto of half the body, 1 ditto for 6 weeks. Feeling of insects crawling over the body, 1 by one trance.—18.

Now we maintain that we are not entitled, in reason, to reject the facts, or alleged facts, above stated without at least a fair examination. It were very easy for us to chime in with the ordinary professional ridicule, in relation to such statements; but, in common honesty, we conceive that we are bound to take a different course. We think it incumbent upon us, fairly to investigate their consistency and authenticity, and to determine the respect or the derision with which they are to be treated, according to their probable truth or falsehood. For the present we shall confine our attention exclusively to the surgical cases; and the first question we have to discuss is this: *Are the statements* FACTS?

In our former article we adduced reasonable evidence to show that cases of coma, unattended by serious or permanent lesion, did sometimes arise spontaneously as disease; that, in such cases, all indications of sensibility were very often absent; and that, as respects a numerous class of mesmeric cases, the only novelty which attaches to them consists in their artificial production. Whether coma and insensibility can or cannot be induced by the mesmeric processes is a matter to be decided by experience only; if we have not ourselves this experience, but rest satisfied with estimating the value of that which is recorded by others, we must, in the formation of a judgment, be guided by the common rules of evidence. In this state of the question we reiterate our former position, that sufficient grounds do actually exist for admitting both the possibility and the probability of coma being artificially produced. The extent to which the statements of particular individuals may justly be credited is, of course, a very different question. But when an honest and intelligent witness circumstantially relates, as having occurred, what is already admitted to be neither impossible nor improbable, the presumption, *a priori*, is certainly altogether in his favour.

In the volume before us we have a well-informed and intelligent surgeon telling us that, within a period of eight months, no less than 73 surgical operations were performed by himself, without there being any indications of suffering on the part of the patients; the whole of the circumstances, moreover, to which reference is made, being abundantly authenticated by respectable names. To suppose the existence of wilful misstatement, on the part of Dr. Esdaile, is out of all question, where detection of falsehood would follow so surely; for our own part we feel confident that, in the instances adduced, there exists, at the very lowest estimate, a *basis* of fact. That our author and his associates should have conspired to delude, and to mystify their friends and their countrymen at home, without any conceivable motive, we hold, indeed, to be too ridiculous to imagine for a moment; and we believe it to be almost as unreasonable to suppose that, in so large a number of instances as those related, individuals should uniformly, as if by common consent, have repressed the very strongest instincts of our nature in avoiding every expression of feeling under agonizing torture,—and that, too, for the mere purpose of deceiving the bystanders. If, however, we could escape the difficulty by admission of this latter explanation, how must we dispose of the first experiments? The earlier patients, who were exclusively of the lowest and most ignorant castes, could certainly know nothing of mesmeric indications; least of all, so completely, as successfully to simulate them. On every sound canon of evidence, then, we must admit the existence of *some* reality in Dr. Esdaile's facts. Let us remember that the statements involve nothing that we should deem to be impossible, even as natural occurrences, for physiological states, analogous to those now under discussion, have arisen spontaneously; besides, well-authenticated instances of insensibility to external impressions have been developed here, in our country, by artificial means; we ourselves can, to a limited extent, testify to this circumstance, as affirmed in our former article. Why, then, should we hesitate to receive Dr. Esdaile's statements with calm respect, and with a disposition to give to them an unprejudiced investigation? It is certainly true that, out of the great number of alleged facts, there are many which are by no means proved to be facts, and not a few which were, in our mind, most probably sheer imposture; we yet reiterate our own conviction that it is most unreasonable to doubt that *some* were *bona fide* cases of artificially induced insensibility. If much of what is here averred were not so very unexpected and wonderful, so much opposed to common notions and every-day experience, and—shall we say?—to our prejudices, it is quite sure that we should never dream of calling it in question.

But although, on many accounts, ready to admit a reality in the reported experience of Dr. Esdaile, as well as in that of a corresponding character nearer home, we would yet caution the reader against the *too facile* reception of this class of facts; to the *proof* of which, undoubtedly, the evidence very often is far from being adequate. It is too common for witnesses in this matter, medical not less than lay, in the first instance to disbelieve altogether in the possibility of inducing, in any degree, states called mesmeric, and then, once satisfied of the contrary, to place almost implicit credence in every statement relating to the subject, however improbable, and however imperfectly attested. As past experience has exhibited cases of temporary insensibility to external impressions, irrespective of mesmerism, and as this fact justly disposes us the more readily to credit the reality of such states, when said to be developed under other circumstances, so, in like manner, past experience has repeatedly displayed instances where the most heroic endurance of pain has enabled the sufferer to repress the outward manifestations of it, and at times, too, for no other purpose apparently than the pure love of deception—a disposition very well known to attack some minds as disease. In judging of the fact of alleged insensibility in particular instances, consideration should of course be always directed to this circumstance. We rarely, however, read mesmeric statements, or see mesmeric experiments, without being struck with the fact of how little evidence, ordinarily, satisfies the all-believing professed mesmerist.

Dr. Esdaile, in the work we have under review, supplies, we think, illustration in some degree of what we have just advanced. He does not appear to us to have been sufficiently aware of the *possibility,* or even the *probability,* of deception,—a circumstance most likely attributable to his having, at the very onset of his career, adopted the general views and the speculations of the animal magnetisers. It is true he states that, prior to his own experiments, his mind was all but a blank to the subject; that he had never seen mesmerism as practised by others; and that he had not even read a mesmeric *book;* but then he acknowledges himself to have read "scraps of newspapers" (most unphilosophical sources of a first impression); he states that he had paid attention to all well-attested reports upon the subject, and that he had ceased to regard it lightly "ever since Dr. Elliotson declared, years ago, that he should despise himself if he did not declare his conviction of the truth of mesmerism." We do but refer to all this as showing that Dr. Esdaile's mind was *not* so divested of prepossession as he himself appears to have imagined; this consideration does not in any way invalidate the statements, but it constitutes a reason for the exercise of caution in determining the *degree* of their credibility. Moreover, there is another circumstance to which we would direct attention: the patients, very generally, were natives of India, and of the lowest and most degraded castes, and sometimes felons. Now it is, we believe, pretty notorious that the Hindoo race is characterised, in a very marked manner, by cunning and deception, and that they have especial satisfaction in deceiving the European; and we should naturally expect these unfavorable traits of natural character to be prominently displayed in the most ignorant and the least moral portion of the community. Neither can we forget the astounding constancy and self-possession with which this race of men endure self-inflicted tortures of the most agonising kind in the exercise of their religious superstitions. Dr. Esdaile does not appear to us to have sufficiently thought of these matters; yet he testifies to the accuracy of the Hindoo characteristic just referred to, where, in speaking of the proceedings of the first French commission of inquiry, he compares D'Eslon to a *Bengal witness,* whom he describes as "not content to tell the truth simply, but added so many corroborating inventions of his own, that no one knew what to believe."

In confutation of the idea that he himself could have been imposed upon by the patients upon whom he had operated, our author remarks as follows:—

"Since then I have had, *every month,* more operations of this kind than take place in the native hospital in Calcutta in a year, and more than I had for the six years previous. There must be some reason for this, and I only see two ways of accounting for it: my patients, on returning home, either say to their friends similarly afflicted, 'Wah! brother, what a soft man the doctor Sahib is! He cut me to pieces for twenty minutes, and I made him believe that I did not feel it. Isn't it a capital joke? Do go and play him the same trick; you have only to laugh in your elbow, and you will not feel the pain.' Or they say to their brother sufferers, 'Look at me; I have got rid of my burthen (of 20, 30, 40, 50, 60, or 80 lbs., as it may be), am restored to the use of my body, and can again work for my bread: this, I assure you, the doctor Sahib did when I was asleep, and I knew nothing about it. You will be equally lucky, I dare say; and I advise you to go and try; you need not be cut if you feel it.' Which of these hypotheses best explains the fact my readers will decide for themselves."

This quotation, we allow, constitutes a very fair method of dealing with the objections of those who would maintain the whole affair to be *humbug*, and that a state of coma and insensibility had in no case been provoked artificially; yet it is very easy to conceive the existence of not very extraordinary *motive* for individuals to affect and to simulate such a condition, *after* the rumours had gone abroad,—in some instances justly,—regarding the painless character of Dr. Esdaile's operations; and the very fact of such rumours having had, *occasionally*, a solid foundation sufficiently accounts for the popularity which the particular hospital obtained. Individuals, from determination not to be outdone, or even from pure love of deceiving others *might* sham. We do not say that this was actually the case; but the possibility—nay, probability—of the thing suggests itself to us on perusal of the book; and we think that the idea is entitled to attention in forming an ultimate estimate of the statements.

The following case, to our minds, is most unsatisfactory; we insert the account as exemplifying what we deem to be the obvious disposition in our author to be satisfied of the genuine character of his facts upon extremely slender evidence:

"August 13th. Dr. Finch freely applied muriatic acid (such as is furnished by the Company's dispensary) to a sore, covering all the right temple of the woman Gendo (who was mesmerised, in his presence, by one of my assistants) without her showing the smallest degree of consciousness; and it was with great difficulty I awoke her, after he had failed to do so. During the burning with the acid her pulse fell from 88° to 80°, and *her respiration, which was quite natural before she was mesmerised, became entirely diaphragmatic and abdominal; the voluntary and semi-voluntary muscles of the chest being completely paralysed.*

"August 18th. Dr. Bedford, to-day, asked permission to apply the acid to the woman Gendo's sore when she was awake; and though I thought this most irrational scepticism (he having witnessed Dr. Finch's experiment) I consented, in order that it might not be said I interfered to save my phenomena. He accordingly wetted the end of the glass stopper with the acid, and touched the sore with it, and *the woman, for a few seconds, did not show any signs of acute pain.* There could be no doubt about it, she was found out! The arch-deceiver, having set a snare and delusion for me, was now laughing at my beard; and I was not relieved from my thick-coming fancies by Dr. Bedford kindly suggesting 'that she was probably a very insensible person naturally.' I was soon roused from my trance of bewilderment, however, by hearing the woman cry out that 'we had put pepper to her head,' and she sat up, showing signs of great pain; immediately after she declared 'her head was on fire,' and got out of bed, walking about distractedly in great agony. I ordered her head to be bathed, and, as the best anodyne, threw her into the trance. The sore being surrounded by tubercles, which retarded its healing, I took the opportunity to pare them off; and to this she was perfectly indifferent. In half an hour I awoke her with much difficulty, in order that Dr. Bedford might hear her first words, which were, that she knew nothing about what we were talking of, having even forgotten the burning."

The change in the character of the respiration, described as occurring "during the burning with the acid" by Dr. Finch, is precisely such as would take place under an effort to conceal the outward signs of suffering. Why, on the acid being applied in the waking state by Dr. Bedford, did not the patient, for some seconds, exhibit indications of pain? Did the manifestations of acute sensibility, after a brief lapse of time, become displayed by the patient, not instinctively, but designedly, just when she discovered that the exhibition was anticipated? Why, moreover, did Gendo retain no recollection of the occurrence after mesmerisation. There was no mesmerism at the time. Was it that the woman was mistaken, in supposing that oblivion in this point of view was expected by the doctors? We refer to these circumstances, because they illustrate the feeble points in not a few of Dr. Esdaile's narratives. Still, we would maintain that, judging of the aggregate of the statements by all the accredited rules of evidence, it is impossible to dispose of this matter of painless operations by the assumption of delusion or imposture in every case.

With respect to the accuracy of the facts relating to the efficacy of mesmerism in the treatment of medical cases, we experience a much greater difficulty in forming a satisfactory decision than we do in the case of the surgical operations; the sources of fallacy, under the former circumstances, being so very much more considerable. From the previous summary given of Dr. Esdaile's recorded experience, it will be perceived that, as is nearly always the case where unwonted methods of cure are said to be employed, diseases of the nervous system form a very large proportion of the group; a class of cases, in which the powerful agency of excited imagination and hope of improvement, howsoever induced, is so universally recognized. Nevertheless, satisfied as we are that abnormal conditions of the system do at times ensue upon the practice of mesmerism, we are quite ready to believe that it may possibly be serviceable in certain nervous affections by exciting a change of action. It is quite certain that, by employment of the "passes" and other means for continuously sustaining uniformity of impression, sleep—common sleep—can at times be procured, when narcotic drugs would either fail or lead to ulterior mischief; and, under such circumstances, it is not difficult to suppose that actual good should arise from use of the means in question. Still, in all these matters, we are ever sceptical concerning the relations of the *post* to the *propter;* for the ordinary records of mesmeric cures always remind us of the obviously mendacious or exaggerated statements attesting the efficacy of every conceivable kind of quackery that has at any time attempted to identify itself with practical medicine. Thus, there is apparently very good evidence to show that epilepsy, hysteria, and neuralgia have sometimes given way under mesmeric treatment; and it frequently seems reasonable enough to attribute the recovery to the means antecedently employed; but then we have just the same evidence to prove that almost every other disease has, at some time or another, been cured by identical processes; nay, the same testimony is adduced to exemplify the curative agency of water about which the "passes" have been made; and sometimes even the therapeutical potency of the mesmeriser's volition is set forth! What wonder if such extravagances throw us back upon our primitive incredulity?

A good many of Dr. Esdaile's mesmeric cures, even as narrated by himself, appear to us to rest absolutely upon no evidence at all, although they are yet cited by himself as undoubted instances of success. The following cases are of this kind:

"June 6th. I was called at eight o'clock last night to see the wife of Baboo Essanchunder Ghosaul, deputy magistrate of Hooghly. I found her in dreadful convulsions; she was speechless, and suffering from a constriction in the throat, that threatened to suffocate her every minute; and she constantly beat or pointed at the part. At one moment her body was perfectly rigid, and in another it was bent back like a bow, till she rested on the back of her head and heels only. I never saw such convulsions except in tetanus and hydrophobia, and all I knew of the resources of medicine was useless; for how could she take physic when she could not take breath? I therefore had recourse to my new solvent power, and after nearly an hour's hard work, I left her asleep and catalepsed.

"July 1st. She has had no return of the fit. This is the lady for whose relief the conjuror was sent, but came too late.

"June 26th. Alunga, aged 24: she has slight contractions of both elbow-joints, from rheumatism, with acute pain on pressing the ulnar nerve at the elbow. At first she did not bear much handling without awaking; but, on being left alone, the trance deepened, and she permitted me to work her joints like door-hinges, and extend them to the natural degree, without awaking. One arm was much freer after the first trance and extension, and there was no pain.

"June 27th. Complains of considerable pain in her left arm to-day, and the nerve at the elbow is very tender. I passed my fingers along the course of the nerve for a few minutes, which removed the pain, and allowed her to extend the arm; I then held my fingers before her eyes for a few seconds, and she fell into my arms insensible.

"July 3d. This woman's pains fly about, but I can chase them away from any part by holding my fingers over it for a short time. She came limping up to me to-day, to have the pain taken out of her 'tendo Achillis;' and this I did by passing my fingers over the pained part. I then grasped it firmly; she felt no pain, and by looks and words expressed the utmost astonishment and delight. This woman's sensibility is such, that I, or any one, can now make her delirious by merely looking at her for five minutes. But more of this hereafter.

"August 18th. I requested Dr. Bedford to satisfy himself if the woman Alunga had pain in any part of her body. On being asked, she said there was acute pain in one heel; and Dr. Bedford spent a long time in testing the reality of its existence. He at last said that he was convinced that there was considerable pain in that spot. I then passed my fingers over the part for a minute, and grasped the heel as firmly as I could, and she declared the pain had vanished; and Dr. Bedford allowed that it had. He then looked at her steadily, and in a few minutes developed the mesmeric delirium, and desire to sleep-walk, always produced in this woman, if the influence is not quickly concentrated upon her. The other symptoms, tremor of the eyelids, inability to open them when closed, and the mesmeric trance, all followed in due course." (pp. 181-4.)

In the first of the above cases we have a somewhat severe attack of hysteria; and, in one hour after the physician's arrival, the paroxysm is over, and the patient goes to sleep. There is nothing to indicate more than the natural termination of the fit; certainly, there is nothing to show that the cessation of the paroxysm could be attributed to the agency employed. But it is exceedingly difficult to deal, in reasoning, with reports of individual facts, as the unavoidable omission of many little circumstances will often interfere with the formation of an accurate judgment with respect to them.

Having, however, fully admitted the high probability of some of Dr. Esdaile's statements concerning the painless character of the surgical operations; and being, indeed, from many circumstances, well convinced that a great depression of outward sensibility, if not its temporary abolition, will, in some constitutions, result from practice of the mesmeric art, we will now proceed to the consideration of what we deem to be the reasonable corollary, from this admission on our own part. We conceive, then, that the evidence attesting the fact of certain abnormal states being induced by mesmerism, is now of such a character that it can no longer be philosophically disregarded by the members of our profession, but that they are bound to meet it in the only way in which alleged facts can satisfactorily be either verified or confuted,—by observation and experiment. When it is positively affirmed that the mesmeric processes will sometimes render a patient utterly insensible to the surgeon's knife, when detailed illustrations of this fact are recorded almost every day, how can we *fairly* reject such statements, unless we go to nature, observe for ourselves, and demonstrate the *source* of the monstrous fallacy that is deluding members of the profession and the public alike? Indeed, we hesitate not to assert that the testimony is now of so varied and extensive a kind, so strong, and in a certain proportion of cases so seemingly unexceptionable, as to authorize us, nay, in honesty, to *compel* us to recommend that an immediate and complete trial of the practice be made in surgical cases. If experience like that which Dr. Esdaile relates to us be but true in one tenth, nay, one hundredth of its particulars, we hold that a case is made out demanding searching inquiry. If mesmerism, even in its humbler pretensions, be absolutely untrue, let it be *proved* to be so. If careful observations and repeated experiment lead to the detection of some hitherto hidden cause of error and mistake that has deluded and mystified the more honest class of mesmerists, what a service will be rendered to humanity and to truth if this can be proclaimed on perfectly just and adequate grounds! In how much better a position shall we be *after investigation* for confuting the imposture, if such it shall turn out ultimately to be, than in continuing to treat the subject with contemptuous disregard! Of one thing let us rest assured, not only the public, but the more sober thinking of the profession will, ere long, hold those at a disadvantage, who, in opposition to *facts*, apparently well authenticated, can or will but adduce mere unsupported argument, or ridicule.

There would appear to be two conditions attaching to any novel practice in medicine, independently of the authority by which it comes recommended, that should influence its title to a fair trial; *first*, the extent of the anticipated benefit, and, *second*, the degree of possible mischief attending its employment. Now the *promised* advantages of mesmerism in surgical operations correspond with these requirements in an eminent degree. If the statements be corroborated, and if insensibility *can* be produced artificially, surely the immense acquisition both to operator and patient is obvious at once; and, according to all the evidence that exists upon this subject, mischief very rarely follows the practice of mesmerism in the event either of success or of failure. "I beg to state," says Dr. Esdaile, "that I have seen no bad consequences whatever ensue from persons being operated on in the mesmeric trance. Cases have occurred in which no pain was felt, even subsequent to the operation, and the wounds healed by the first intention; and in the rest I have seen no indication of any injurious consequences to the constitution. On the contrary, it appears to me to have been saved, and that less constitutional disturbance has followed than under ordinary circumstances." If then *good* is possibly to ensue, and *mischief* is but little to be feared from the experiment, why not candidly make it? Assuredly experiments in therapeutics are constantly made on grounds far less reasonable. If a single practitioner of any eminence recommend some novel and *heroic* treatment in serious disease, multitudes are ready to try it; however perilous to the patient the trial, *a priori*, may appear. Although, at the present day, it is pretty well made out that pneumonia, in many instances, will come to a successful issue with very little depletion, some dozen years since large numbers of the profession, especially in France, did not hesitate, on the recommendation of M. Bouillaud, to bleed *coup sur coup;* and, about twenty years ago, when Dr. Armstrong bled largely, and administered heroic doses of calomel in the incipient stage of fever, many persons felt themselves authorized in adopting the treatment experimentally. Yet, in these instances, a degree of risk to the patient was incurred in the attainment of the possible benefit, and there was, moreover, an uncertainty in deciding upon the exact nature of the result, which, as regards mesmerism in surgery, would not be experienced. Again, we say, let it be *tried* upon patients about to be submitted to the knife; if true, let us have the benefit of it, and if false let the falsehood be *demonstrated.*

Experiments relating to the treatment of medical diseases by any doubtful remedy could not result in the same decided issue, as in the example of mesmerism applied in operative surgery. We may observe, however, that when full satisfaction has been obtained respecting the efficacy of certain processes in the production of coma and insensibility, it would naturally be expected that, in various morbid states, the induction of such conditions may aid the restorative powers of the system; at any rate, in the present acknowledged paucity of positive therapeutical agents in numerous diseases, especially in those prominently affecting the nervous functions, the trial might very reasonably be made. We think sufficient evidence exists to prove the power of the mesmeric passes—or, we would rather say, the passes of mesmerists—as also of Mr. Braid's hypnotic fascination, in occasionally conciliating sleep, in nervous cases, after the failure of ordinary medical means. We may attach no importance to the *mesmerism* employed in such cases as involving specific agency; the patients may have been made to sleep very likely by the process operating as an appropriate *lullaby,* and the improvement have thus followed. It is, however, satisfactory to know that, under such circumstances, we can sometimes provoke sleep, and thereby effect a mitigation of human suffering.

We have before said that Dr. Esdaile exhibits a full and undoubting faith in most of the phenomena usually recorded as characterizing mesmerism; and thus, in reasoning upon the assumed resultant benefit in medical cases, he constantly avails himself of the theory of animal magnetism. In speaking of the curative agency, he holds it to be due to the transference of a vital fluid from the person of the mesmeriser to that of the mesmerised. We have too recently devoted a portion of our pages to this point, to reopen the discussion upon the present occasion; we can only observe that, after a careful perusal of Dr. Esdaile's book, we do not see that he has strengthened one whit the idea of exoteric animal influence—of objectivity—in mesmerism. We do not *deny* the reality of animal magnetism; we assert, however, that it is not *proved,* at least to our satisfaction.

In dealing with the subject of mesmerism, we are but desirous of coming at the truth: we have already given our reasons for thinking that the time is come when, so far as it may be false, it can only be put down by calm and unprejudiced inquiry. From the curious and extraordinary accumulation of records amassed by the animal magnetisers, we have from the beginning been very much persuaded that, at the foundation of all the extravagances of the mesmeric disquisitions, there would ultimately be discovered *some* truth. So far back as the year 1839, after presenting to the reader a brief historical account of animal magnetism, we stated that we could not "be so far influenced by the impostures occasionally practised under the name of magnetism, as wholly to deny that some of the phenomena, from time to time produced by all aspirers to the art, seem to result either from some principle heretofore unknown and not yet correctly designated, or from some modification of recognized principles in the animal economy, which cannot yet be accurately limited or defined." In our article of last year, we entered into a more careful investigation of the particular facts, attempting some discrimination of the most probably true from the almost certainly false. In the former category we included the artificially produced *sleep, coma, altered sensibility, spasm, or temporary paralysis of muscles, &c.*—THE SIMPLE PHENOMENA OF MESMERISM; our admission of the reality of these conditions rested, *first*, upon their antecedent probability, as being, in most respects, analogous to abnormal states sometimes arising spontaneously as disease; *secondly*, upon the fact of the evidence attesting them corresponding with all just requirements; and, *lastly*, on the circumstance of having ourselves either seen them or been grossly duped in spite of a very rigid scrutiny. In like manner—exactly on the same grounds—we admitted the reality of mesmeric *somnambulism*, to the exclusion of *clairvoyance* and the other phenomena comprehended in the term *lucidity*. These latter, however, as well as the theory of animal magnetism, we only rejected as *unproved;* because, unlike the simple mesmeric phenomena so designated, we had sought for the reality in vain, and because the evidence, when fully and fairly tested, had always appeared to us to break down—feeble and inconclusive at the best. Within the last eighteen months we have not been indifferent spectators of what has been going on with respect to these matters; we have read, and we have ourselves investigated, with minds open to conviction and indifferent to every consideration but that of *truth*, and our conclusions remain unchanged. We must reiterate that, in our judgment, after making all due abstraction of the roguery and deceit notoriously prevalent in many mesmeric exhibitions, there is but a reality in the simple phenomena as just enumerated; that the induction of these does not necessarily imply the existence of exoteric animal influence; but that, as formerly maintained by M. Bertrand in France, and still more recently by Mr. Braid in this country, the mesmeric states most probably, in all cases, arise from sensible—not occult—impressions; and that the higher phenomena, which imply the receipt of intelligence otherwise than through the customary channels, are not only in the highest degree improbable, but are utterly unsupported by the requisite force of evidence.

